# Complement gene variants in relation to autoantibodies to beta cell specific antigens and type 1 diabetes in the TEDDY Study

**DOI:** 10.1038/srep27887

**Published:** 2016-06-16

**Authors:** Carina Törn, Xiang Liu, William Hagopian, Åke Lernmark, Olli Simell, Marian Rewers, Anette-G Ziegler, Desmond Schatz, Beena Akolkar, Suna Onengut-Gumuscu, Wei-Min Chen, Jorma Toppari, Juha Mykkänen, Jorma Ilonen, Stephen S. Rich, Jin-Xiong She, Ashok Sharma, Andrea Steck, Jeffrey Krischer, The TEDDY Study Group, Michael Abbondondolo, Janey Adams, Annika Adamsson, Daniel Agardh, Stephen W. Anderson, Carin Andrén Aronsson, Maria Ask, Sarah Austin-Gonzalez, Stephen Ayres, Sandra Baethke, Kimberly Bautista, Judith Baxter, Dorothy Becker, Ruth Bedoy, Rasmus Bennet, Suzanne Bennett Johnson, Andreas Beyerlein, Ezio Bonifacio, Kasia Bourcier, Jenny Bremer, Thomas Briese, Rasheedah Brown, Brant Burkhardt, Martha Butterworth, Ulla-Marie Carlsson, Corrado Cilio, Joanna Clasen, Claire Cowen Crouch, David Cuthbertson, Ashi Daftary, MaryEllen Dalmagro-Elias, Kayleen Dunson, Christopher Eberhard, Helena Elding Larsson, Emelie Ericsson-Hallström, Daniel Felipe-Morales, Steven Fiske, Gabriella Foghis, Kristina Foterek, Margaret Fransiscus, Lina Fransson, Brigitte I. Frohnert, Dena Garcia, Thomas Gard, Melissa Gardiner, Jennifer Garmeson, Joanna Gerardsson, Patricia Gesualdo, Veena Gowda, Michael Haller, Monica Hansen, Gertie Hansson, Cecilia Harmby, Rachel Hervey, Kathleen Heyman, Michelle Hoffman, Diane Hopkins, Michael Hummel, Sandra Hummel, Susanne Hyberg, Heikki Hyöty, Fredrik Johansen, Corbin Johnson, Sanna Jokipuu, Berglind Jonasdottir, Tiina Kallio, Rachel Karban, Mathilde Kersting, Michael Killian, Beth Klein, Mikael Knip, Annette Knopff, Annika Koivu, Sibylle Koletzko, Mirva Koreasalo, Kalle Kurppa, Miia Kähönen, Hye-Seung Lee, Sigrid Lenrick Forss, Edwin Liu, Shu Liu, Markus Lundgren, Kristian Lynch, Rachel Lyons, Maria Lönnrot, Jamie Malloy, Maria Markan, Cristina McCarthy, Richard McIndoe, Wendy McLeod, Jessica Melin, Zeliha Mestan, Steven Meulemans, Arlene Meyer, Denise Mulenga, Katja Multasuo, Maria Månsson-Martinez, Elina Mäntimäki, Tiina Niinien, Jill Norris, Mia Nyblom, Claudia Peplow, Francisco Perez Laras, Kobra Rahmati, Petra Rajala, Anita Ramelius, Jenna Rautanen, Anne Riikonen, Richard Robinson, Minna Romo, Anna Rosenquist, Roswith Roth, Falastin Salami, Adela Samper-Imaz, Elisabeth Scott, Chris Shaffer, Sara Sibthorpe, Katherine Silvis, Satu Simell, Ville Simell, Maija Sjöberg, Birgitta Sjöberg, Jennifer Skidmore, Laura Smith, Susan Smith, Joshua Stabbert, Leigh Steed, Aino Stenius, Joanna Stock, Elisabeth Strauss, Noah Sulman, Ulrica Swartling, Maria Särmä, Roy Tamura, Alexander Tarr, Evelyn Tekum Amboh, Jamie Thomas, Eric Triplett, Erika Trulsson, Morgan Uland, Ulla Uusitalo, Sini Vainionpää, Anne Wallin, Eeva Varionen, Katharina Warncke, Kathleen Waugh, Kendra Vehik, Riitta Veijola, Ponni Vijayakandipan, Joshua Williams, John Willis, Åsa Wimar, Christiane Winkler, Suvi M. Virtanen, Keith Wood, Hali Wright, Mari Vähä-Mäkilä, Jimin Yang, Chrystal Yates, Sofie Åberg, Mari Åkerlund

**Affiliations:** 1 Department of Clinical Sciences, Lund University/CRC, Malmö, Sweden; 2 Health Informatics Institute, Morsani College of Medicine, University of South Florida, Tampa, FL, USA; 3 Pacific Northwest Diabetes Research Institute, Seattle, WA, USA; 4 Department of Pediatrics, Turku University Hospital, Turku, Finland; 5 Barbara Davis Center for Childhood Diabetes, University of Colorado, Aurora, CO, USA; 6 Institute of Diabetes Research, Helmholtz Zentrum, München, and Klinikum rechts der Isar, Technische Universität München, and Forschergruppe Diabetes e. V., Neuherberg, Germany; 7 Department of Pediatrics, University of Florida, Gainesville, FL, USA; 8 National Institutes of Diabetes & Digestive & Kidney Disorders, Bethesda, MD, USA; 9 Center for Public Health Genomic, University of Virginia, Charlottesville, VA, USA; 10 Departments of Physiology and Pediatrics, University of Turku, Turku, Finland; 11 Center for Biotechnology and Genomic Medicine, Medical College of Georgia, Augusta University, Augusta, GA, USA; 12 Turku University Hospital, Hospital District of Southwest Finland, Turku, Finland; 13 Children's Hospital of Pittsburgh of UPMC, Pittsburgh, PA, USA; 14 Florida State University, Tallahassee, FL, USA; 15 Center for Regenerative Therapies, TU, Dresden, Germany; 16 National Institutes of Allergy and Infectious Diseases, Bethesda, MD, USA; 17 Columbia University, New York City, NYC, USA; 18 Research Institute for Child Nutrition, Dortmund, Germany; 19 University of Tampere, Tampere, Finland; 20 Tampere University Hospital, Tampere, Finland; 21 Dr. von Hauner Children’s Hospital, Department of Gastroenterology, Ludwig Maximilians University Munich, Germany; 22 National Institute for Health and Welfare, Finland; 23 University of Oulu, Oulo, Finland; 24 Oulo University Hospital, Oulo, Finland; 25 University of Florida, Gainesville, FL, Florida, USA

## Abstract

A total of 15 SNPs within complement genes and present on the ImmunoChip were analyzed in The Environmental Determinants of Diabetes in the Young (TEDDY) study. A total of 5474 subjects were followed from three months of age until islet autoimmunity (IA: n = 413) and the subsequent onset of type 1 diabetes (n = 115) for a median of 73 months (IQR 54–91). Three SNPs within *ITGAM* were nominally associated (p < 0.05) with IA: rs1143678 [Hazard ratio; HR 0.80; 95% CI 0.66–0.98; p = 0.032], rs1143683 [HR 0.80; 95% CI 0.65–0.98; p = 0.030] and rs4597342 [HR 1.16; 95% CI 1.01–1.32; p = 0.041]. When type 1 diabetes was the outcome, in DR3/4 subjects, there was nominal significance for two SNPs: rs17615 in *CD21* [HR 1.52; 95% CI 1.05–2.20; p = 0.025] and rs4844573 in *C4BPA* [HR 0.63; 95% CI 0.43–0.92; p = 0.017]. Among DR4/4 subjects, rs2230199 in *C3* was significantly associated [HR 3.20; 95% CI 1.75–5.85; p = 0.0002, uncorrected] a significance that withstood Bonferroni correction since it was less than 0.000833 (0.05/60) in the HLA-specific analyses. SNPs within the complement genes may contribute to IA, the first step to type 1 diabetes, with at least one SNP in *C3* significantly associated with clinically diagnosed type 1 diabetes.

Currently, autoantibodies are the best markers for screening purposes in large populations of an autoimmune attack directed towards the pancreatic islet beta cells. Although the attack is thought to be primarily carried out by cytotoxic T cells that recognize autoantigen peptides on beta-cell HLA Class I proteins it cannot be excluded that other components of the innate immune system also contributes. Complement factors are proteins involved in the innate immune system assisting in opsonizing foreign antigens to increase the immune attack towards bacteria and viruses. Other functions of the complement system include chemotaxis where macrophages and neutrophils are attracted to the site of infection, cell lysis which is a rupture of foreign cells and agglutination where pathogens are clustered together. Complement factors are also linked to the adaptive immune system since antibodies form complexes with complements. Genetic variants of genes encoding complement factors have shown associations with autoimmune diseases, in particular to type 1 diabetes, systemic lupus erythematosus (SLE) and autoimmune macular degeneration (MAD). In type 1 diabetes, studies have shown an increased deposition of C4 in pancreata^1^, as well as increased levels of plasma protein inhibitor (C1-inhibitor)^2^. Deposition of C5b-9 (the membrane attack complex (MAC) or terminal complement complex (TCC)) has been identified in neurons of those with type 1 diabetes who later died from ketoacidosis^3^. Increased innate immune reactivity has been detected before diabetes-associated seroconversion in children with high-risk HLA genotypes, for example an increased expression of C2 and C4 binding protein alpha-chain (C4BPA) one to two years prior to diagnosis of type 1 diabetes^4^. Human serum may be toxic to beta-cells by activating the alternative complement pathway^5^.

Four genes encoding complement proteins (factor B, C2, C4A and C4B) are located on chromosome 6 in the human major histocompatibility complex (MHC) that also contains the type 1 diabetes associated HLA-genes. The complement genes (*CFB, C2, C4A, C4B*) are located telomeric between the HLA class II and class I regions. Given the extensive linkage disequilibrium in the MHC, these complement genes are inherited as a fixed set (a complotype) and have been included in the HLA cluster as an extended HLA haplotype. At least 15 different complotypes have been identified with frequency greater than 1% within Caucasian populations^6^. Certain haplotypes within the HLA- complement system are associated with type 1 diabetes, particularly those that include HLA-DR3-DQ2. The extended haplotypes of HLA and complement (HLA-A1-B8-BfS- C4AQ0-DR3, HLAB18-BfF1-C4A3-C4BQ0-DR3-DQ2 and HLA-A2-CW3-BW62-BfS-C4A3-DR4-DQ8) are increased among type 1 diabetes cases compared with controls^7^. These three haplotypes have been replicated many times as well as three additional haplotypes exhibiting association with type 1 diabetes. The B7-BfS-DR2 haplotype has negative association with type 1 diabetes (more common in controls than in cases), while five are risk haplotypes of type 1 diabetes, more common in patients than in controls (B8-BfS-DR3, B8-BfS-DR4, B15-BfS-DR4, B18-BfF1-DR3 and B40-BfS-DR4)^8^. Whether these complement gene variants are themselves affecting susceptibility to type 1 diabetes or are in linkage disequlibrium with other risk contributing genes (including certain HLA class I alleles) remains unresolved^9^,^10^,^11^.

There are three different pathways that activate complement: the classical pathway (immunoglobulin bound to antigen), the mannose binding lectin pathway and the alternative pathway. The initial enzyme in the classical pathway is C1 (C1q/C1r/C1s). C1q can bind to IgG molecules leading to transactivation of C1q. C1s in turn cleaves the proteins C4 and C2 into C4a and C2b. The formation of C4b2a acts as the C3 convertase activating enzyme in the classical pathway. The mannose binding lectin pathway (mannose binding to lectin/ficolin bound to carbohydrate on pathogen), starts with recognition via mannose-binding lectin (MBL), ficolin 1–3 and CL 11, the process continues with involvement of mannose-binding lectin associated serine proteases (MASP-1, MASP-2 and MASP3) leading to cleavage and activation of C4, C2 and C3. The alternative pathway is initiated by presence of pathogen leading to a spontaneous activation of C3. The three pathways results in the formation of C3 convertases that cleave C3 to the C3b and C3a forms. Thereafter C5 is activated and together with C6–9 form the C5b9 (MAC)^12^,^13^. It has been shown that several Ig classes (IgG, IgM and IgA) can be involved in the three pathways^13^.

Approximately, 50 SNPs within complement genes have been associated with different autoimmune diseases. On the ImmunoChip, a custom genotyping array based upon significant genome-wide association with autoimmune loci with autoimmune diseases, there are 17 complement SNPs that can be interrogated in the TEDDY study.

The Environmental Determinants of Diabetes in the Young (TEDDY) is an international prospective study of newborn children with an increased genetic risk for type 1 diabetes. Children from the general population were enrolled into TEDDY based on their increased genetic risk to type 1 diabetes based upon any of the four high-risk HLA-genotypes (DR3/4, DR4/4, DR4/8 or DR3/3). The goal of TEDDY is to identify environmental factors that precipitate islet autoimmunity (IA) and subsequent type 1 diabetes in genetically predisposed children. Type 1 diabetes develops in at least two phases. A hallmark of the first phase, is an unknown event or agent that may trigger the immune system to produce autoantibodies against either insulin, GAD65 or IA-2, alone or in combinations as an expression of IA. In this first subclinical phase, there is a shortcoming of insulin production. In the second phase, increased blood glucose levels can be detected if an oral glucose tolerance test (OGTT) is performed^14^. This second phase ends with an infiltration of cytotoxic T- cells into the islets of Langerhans’^15^. Persistent confirmed IA, defined as positivity for an autoantibody on two consecutive visits and confirmed in a second laboratory, is the primary outcome of TEDDY. The secondary outcome in TEDDY is the diagnosis of type 1 diabetes as defined by the American Diabetes Association^16^. It is well documented that the HLA-region contributes the greatest proportion of genetic risk (~50%) for type 1 diabetes^17^. Several other genes within the immune system also contribute to the genetic susceptibility^18^,^19^ in combination with environmental factors.

In the present study, the primary aim was to determine whether SNPs within complement factors were associated with IA, or type 1 diabetes, or both, in TEDDY children.

## Results

As of 31 December 2014, 7.5% (413/5474) children developed any persistent confirmed IA (M/F = 1.43; 243/170). A total of 2.1% (115/5474) of high risk children had progressed to type 1 diabetes (M/F = 1.30; 65/50); however 5 of the 115 who converted to type 1 diabetes did not have IA. The median follow-up time was 73 months (IQR 54–91). The median age at seroconversion to persistent IA was 27.9 (IQR: 15.4–47.8) months and the median age at onset of type 1 diabetes was 51.4 (IQR: 29.2–67.5) months. Tables 1 and 2 summarizes demographic characteristics of the subjects who developed IA and those who developed type 1 diabetes. The three HLA-genotypes including DR4 all conferred increased hazard ratios using the time to develop IA as compared with DR3/3; DR4/8 [HR = 1.57; 95% CI 1.08–2.28; p = 0.018], DR4/4 [HR = 1.74; 95% CI 1.22–2.47; p = 0.002] and in particular DR3/4 [HR = 2.37; 95% CI 1.75–3.22; p < 0.001]. The high-risk genotype HLA-DR3/4 conferred an increased hazard ratio for time to develop type 1 diabetes as compared with DR3/3 [HR = 2.28; 95% CI 1.30–4.00; p = 0.004].

### Hazard ratios using the time to IA in relation to SNPs in complement genes

Proportional hazard modeling identified three SNPs as nominally associated (p < 0.05, prior to Bonferroni correction) with IA in the presence of high-risk HLA genotypes in TEDDY participants. Two SNPs in integrin alpha M (*ITGAM)* were more common in controls than cases (protective): rs1143678 [HR = 0.80; 95% CI 0.66–0.98; p = 0.032] and rs1143683 [HR = 0.80; 95% CI 0.66–0.98; p = 0.030].

SNP rs4597342 in *ITGAM* was associated with an increased hazard ratio using time to IA [HR = 1.16; 95% CI 1.01–1.32; p = 0.041] (Table 3).

After Bonferroni correction, none of the 15 SNPs remained as significant.

There was no significant interaction between any of the complement SNPs on IA and HLA- risk categories.

### Hazard ratio using time to type 1 diabetes in relation to complement SNPs

None of the 15 SNPs tested was associated with time-to-type 1 diabetes in the entire cohort (Table 4).

**Figure 1:**
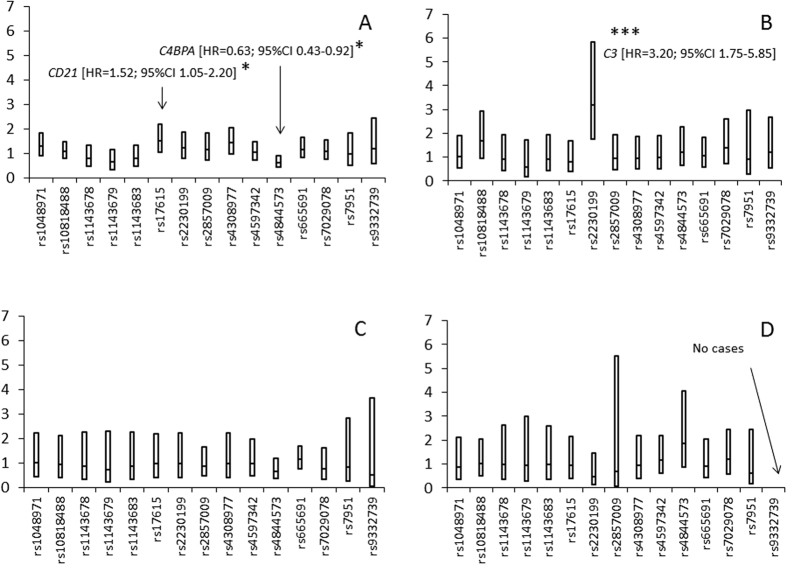
The figure illustrates a summary of 15 SNPs within genes in the complement system and present on the ImmunoChip that were interrogated in The Environmental Determinants of Diabetes in the Young (TEDDY) study in 115 subjects that had developed type 1 diabetes versus all other subjects (n = 5359). Subjects eligible for TEDDY carried any of the four high risk HLA-DR-DQ genotypes (DR3/4, 4/4, 4/8 or 3/3). The upper 95% confidence interval (CI) is indicated by the upper bar of the box, the lower. 95% CI is indicated by the lower bar of the box and the hazard ratio (HR) is indicated by a solid bar in the middle of the box. Three hazard ratios were different from 1.0 but only rs2230199 in *C3* (p = 0.0002) among the DR4/4 carriers remained significant after Bonferroni correction since it was less than 0.000833 (0.05/60) in the HLA specific analyses. The explicit values of the HR (95% CI) for type 1 diabetes in the HLA stratified analyses can be found in an online Appendix. Panel A HRs and 95% CIs in 65 subjects with type 1 diabetes and 2139 subjects without type 1 diabetes carrying the HLA-DR3/4-genotype (n = 2204). Panel B HRs and 95% CIs in 19 subjects with type 1 diabetes and 1067 subjects without type 1 diabetes carrying the HLA DR4/4-genotype (n = 1086). Panel C HRs and 95% CIs in 16 subjects with type 1 diabetes and 950 subjects without type 1 diabetes carrying the HLA-DR4/8-genotype (n = 966). Panel D HRs and 95% CIs in 15 subjects with type 1 diabetes and 1203 subjects without type 1 diabetes carrying the HLA- DR3/3- genotype (n = 1218).

There were two significant interactions of SNPs with HLA genotype on type 1 diabetes; rs2230199 in *C3* (p = 0.0125) and rs4844573 in *C4BPA* (p = 0.0275). When the cohort was stratified based on HLA- risk- category, three SNPs achieved nominal significance in the time-to-event analysis. Among DR3/4 subjects SNPs: rs17615 in *CD21* (C3d-receptor) [HR = 1.52; 95% CI 1.05–2.20; p = 0.025: n = 2204] and rs4844573 in *C4BPA* [HR = 0.63; 95% CI 0.43–0.92; p = 0.017: n = 2204] reached p < 0.05 (Fig. 1A). Among DR4/4 subjects SNP rs2230199 in *C3* [HR = 3.20; 95% CI 1.75–5.85]; p = 0.0002; n = 1085] achieved Bonferroni-corrected significance since it was less than 0.000833 (0.05/60) in the HLA-specific analyses (Fig. 1B). There were no significant associations for any of the 15 SNPs with respect to type 1 diabetes among DR4/8 or DR3/3 subjects (Fig. 1C,D).

## Discussion

In the present study we were not able to demonstrate a robust association between IA and SNPs within the complement genes. Nevertheless, we found a significant association with type 1 diabetes time-to-event with one SNP (rs2230199) in the *C3* gene, but only among DR4/4 subjects. There was a strong association for *C3* rs2230199 among HLA-DR4/4 carriers, the genotype conferring the second highest risk for type 1 diabetes. An infection could precipitate type 1 diabetes in those with genetic predisposition (based upon rs2230199 in *C3*) since the expression levels of *C3* could be different and these subject would not be able to vigorously fight the pathogen. *C3* is a key player in the formation of MAC from all three pathways in the activation of complements.

An infection within the islets of Langerhans’ shortly before the onset of type 1 diabetes could explain the finding of cytotoxic T- cells, which are part of the innate immune response, at the time of diagnosis. The *C3* rs2230199 SNP is a functional polymorphism, where the G allele causes an amino acid substitution where arginine is substituted with glycine (R102G)^20^. The glycine variant of *C3* moves faster in electrophoresis and has been shown to confer increased adhesion to monocytes^21^. The R102G variant has shown a strong association with age-related macular degeneration (AMD)^20^,^22^,^23^. One feature indicating different *C3* levels in those with type 1 diabetes is that children with type 1 diabetes have impaired clot lysis due to increased incorporation of *C3* into clots thereby prolonging lysis time^24^.

There was a strong correlation between the three SNPs in *ITGAM* (rs1143678 *vs* rs1143679, r_s_ = 0.85, p < 0.0001, rs1143678 *vs* rs1143683, r_s_ = 0.99, p < 0.0001 and rs1143679 *vs* rs1143683, r_s_ = 0.85, p < 0.0001) consistent with strong linkage disequilibrium in this region. All three SNPs have previously been shown to impair Mac-1-mediated neutrophil phagocytosis and phagocytosis mediated by Fcγ receptors as well as to impair the firm adhesion of neutrophils in healthy subjects^25^. SNP rs17615 in *CD21* was associated with an increased hazard ratio using time to type 1 diabetes among HLA-DR3/4 carriers, while those with the rs17615 minor allele have been shown to have a protection from SLE. SNP rs17615 (together with rs1048971 and rs4308977) have a biological function that decrease the splicing of the alternatively spliced exon 11 conferring increased amounts of the short CR2 isoform^26^. *ITGAM*, also denoted CD11b-integrin is an adhesion molecule expressed on several cells within the innate immune system such as granulocytes, monocytes, macrophages and natural killer cells. Moreover, *ITGAM* is involved in the complement system due to its capacity to bind inactive complement component 3b (iC3b)^27^. One variant within *ITGAM* (rs1143679, A-allele) confers an amino acid substitution where arginine is substituted with histidine R77H. This substitution causes a functional dysfunction of *ITGAM* leading to impaired adhesion and reduced phagocytosis of complement opsonized antigens. Interestingly, the R77H variant of *ITGAM* is highly associated with systemic lupus erythematosus (SLE)^28^. *CD21* is also known as the C3d-receptor or the Epstein-Barr virus receptor^29^. The C3d-receptor is expressed on B-cells and has been shown to increase the B-cell receptor (BCR) signaling as well as to enhance the processing of antigens^30^. *C4BPA* has an inhibitory effect within the complement system^31^.

The major strength of the present study is that we have children followed from birth who, in some cases, developed IA and in some cases progressed to type 1 diabetes, thereby allowing us to study both disease initiation and progression. Additional strengths are that we only have included subjects that have high-risk HLA genotypes, thereby setting an equal baseline for the major genetic susceptibility. The study design with repeated sampling (more often in the toddlers and more dispersed after four years of age) allowed us to use proportional hazard ratio modeling to identify hazard ratios for time to end-points (IA and type 1 diabetes) in this cohort consisting of three European populations and a USA population (three centers combined). This study design increase the power compared with cross-sectional studies of cases and controls. A current limitation on the study, is the relatively low number of participants that have reached the end-points (IA and type 1 diabetes). The Bonferroni correction might be too conservative if some SNPs are in linkage disequilibrium as is most likely the case for the four SNPs within *ITGAM*, the three SNPs in *CD21* the two SNPs in *C3* and the two SNPs in *C5*. Nevertheless, among DR4/4 subjects SNP rs2230199 in *C3* achieved Bonferroni-corrected significance. As the predisposition to type 1 diabetes occurred only in those carrying HLA-DR4/4, the proposed apparent contribution is necessary and requires confirmation in functional studies.

As our TEDDY cohort gets older more children will proceed to IA and type 1 diabetes, and we will have more power in our determination of association of complement factor gene variants and hazard ratios using time to IA and type 1 diabetes.

In summary, although complement gene SNPs are not robustly associated with IA or type 1 diabetes, we concluded that variants in the complement genes do have some apparent contribution to IA and type 1 diabetes. However, discrimination of complement pathways effects requires further research.

## Subjects and Methods

Newborn children were screened for high-risk HLA genotypes and enrolled into the TEDDY study from 1 September 2004 until 28 February 2010. A total of 424 788 newborns in Finland, Sweden, Germany and the United States (Colorado, Georgia and Washington) were screened as previously described^32^,^33^. The HLA high- risk genotypes for subjects from the general population (GP) were as follows: DR3/4, DR4/4, DR4/8 and DR3/3. An additional six genotypes were used for first degree relatives (FDRs) to a subject with type 1 diabetes: DR4/4, DR4/1, DR4/13, DR4/4, DR4/9, DR3/9 (Table 5). DR4 subtyping was performed to exclude children from the GP with DRB1*04:03. DQB1*03:04 also qualified for inclusion into the TEDDY study. Subtyping was not done to distinguish BQB1*02:0X and DQA1*03:0X subtypes. In the DQB1*05:01 haplotype, DR10 had to be excluded, only DR1 was eligible. A total of 8667 newborn children were enrolled based on these criteria.

The initial blood sample for HLA- screening was obtained either as cord blood in the maternity clinic or as dry blood spot (DBS) on day three to four. If the child carried any of the high- risk genotypes, the family was contacted by a study nurse and invited to participate in the 15 year follow-up study with blood sampling for analysis of autoantibodies (GADA, IA-2A and mIAA) every 3 months between 3 and 48 months of age and every six months thereafter. HLA- genotypes were confirmed in a second blood sample at the 9- month visit. This blood sample was also used for the ImmunoChip SNP- analyses. This study was performed according to the principles expressed in the Declaration of Helsinki. Signed informed consents were obtained for all study participants from their parents or primary caretakers, separately, for genetic screening and participation in the follow-up. The study was approved by local Institutional Review Boards and is monitored by an external evaluation committee formed by the National Institutes of Health. The local Institutional Review Boards were as follows: Colorado Multiple Institutional Review Board (Colorado), Ethics Committee of Southwest Finland Hospital District (Finland), University of Florida Health Center Institutional Review Board, Medical College of Georgia Human Assurance Committee (2004–2010)/Georgia Health Sciences University Human Assurance Committee (2011–2012)/Georgia Regents University Institutional Review Board (2013–present) (Georgia), Bayerischen Landesärztekammer Ethics Committee (Germany), Regional Ethics Board in Lund, Section 2 (2004–2012)/Lund University Committee for Continuing Ethical Review (2013–present) (Sweden), Washington State Institutional Review Board (2004–2012)/Western Institutional Review Board (2013–present) Washington.

### Study outcome – Islet autoimmunity (IA) and type 1 diabetes

The primary outcome was the finding of persistent confirmed IA assessed every 3 months between 3 and 48 months of age and every 6 months thereafter. IA was confirmed if identified in both Reference Laboratories. Persistent autoimmunity was defined by the finding of confirmed IA (any of GADA, IA-2A or mIAA) on two or more consecutive visits. Date of persistent confirmed IA was defined as the draw date of the first of two consecutive samples of at which the child was confirmed as “positive” for a specific autoantibody or any autoantibody. Because children can be born with maternal islet autoantibodies, positive results due to maternal transmission via placenta were excluded when defining the child’s IA status. In order to discriminate maternal autoantibodies from IA in the child, the IA status of the mother was assessed when the child was 9 months if IA was detected at 3 or 6 months of age. The child’s IA status was defined based on both maternal and child IA. If maternal autoantibodies were identified, the child was not considered persistently IA positive unless the child had a negative sample prior to the first positive sample. All samples with a positive result of IA and 5% of negative samples were re-analyzed for confirmation in both Reference Laboratories. In the U. S., all samples were assayed at the Barbara Davis Center for Childhood Diabetes at the University of Colorado, Denver; in Europe all samples were assayed at the University of Bristol, the U. K. All the autoantibodies were measured using radioimmuno-binding assays^34^,^35^,^36^,^37^. The secondary outcome of the study was the diagnosis of type 1 diabetes as defined by guidelines from the American Diabetes Association^16^.

### HLA- typing

HLA- genotype screening was performed using either a DBS punch or a small volume whole blood lysate (WBL)^33^,^38^. Following PCR amplification of exon 2 of the HLA class II gene (DRB1, DQA1 or DQB1), alleles were identified either by direct sequencing, oligonucleotide probe hybridization, or other genotyping techniques. Typing to certify specific DR-DQ haplotypes were specified for each clinical center. HLA genotypes in eligible subjects were confirmed, using the 9-month sample, by the central HLA Reference Laboratory at Roche Molecular Systems, Oakland, CA.

### Single Nucleotide Polymorphisms (SNPs)

SNP analysis was performed by the Center for Public Health Genomics at University of Virginia, using the Illumina ImmunoChip. The ImmunoChip is a custom array for genotyping of SNPs selected from regions of the human genome firmly associated with autoimmune diseases. The final selection of SNPs containing ~186 000 SNPs in 186 regions, for 12 autoimmune diseases was decided by the ImmunoChip Consortium. The 9- month sample was used for SNP genotyping after DNA extraction done by Roche Molecular Systems (Pleasanton, CA). Quality control (QC) steps to assure high quality of the reported SNPs comprised the exclusion of subjects due to low call rate (>5% SNPs missing) and discordance with reported sex and prior genotyping. Secondly, SNPs were removed from analysis due to low call rate (<95%), Hardy-Weinberg equilibrium (HWE) p-value <10^−6^ (except for chromosome 6 due to HLA eligibility requirements) as well as being monomorphic or an insertion-deletion.

A total of 17 SNPs residing in loci within genes coding for complement factors were present on the ImmunoChip. Of these 17 SNPs, two did not pass QC (rs1061170 in *CFH* and rs2274567 in *CD35*). In the TEDDY study, we have previously performed a confirmation study of 41 SNPs associated with type 1 diabetes and risk of autoantibody positivity^39^.

### Study population

To allow more generalizable interpretations of the genetic associations, we included non-Hispanic Whites from the US sites and all participants from the European sites. Only participants identified without a type 1 diabetes -FDR were included. After applying these criteria, a total of 5474 participants (M/F = 1.06; 2817/2657) with data on 15 SNPs remained in the study.

### Statistical analyses

Two outcomes were analyzed: the time to persistent confirmed IA and the time to type 1 diabetes. The time to persistent confirmed IA was defined as the age when the first of confirmed positive samples was taken, and the right censored time when the last negative sample was taken. The time to type 1 diabetes was the child’s age at the time of diagnosis of type 1 diabetes. The primary analysis was to investigate the association of the SNPs within the complement genes with the risk of development of persistent confirmed IA/type 1 diabetes. The minor allele frequency (MAF) was determined in the selected cohort (n = 5474) and used throughout the analyses. This strategy infers that the risk is associated with the minor allele, even though the certain allele that has the lower frequency may vary across populations. Cox proportional hazard model was used for the analyses, adjusting for HLA, sex and country of residence. In addition, the first two principal components (PC) of the ImmunoChip data were used as covariates in the Cox model to adjust for potential population stratification. The axes of the principal components were estimated based on the TEDDY US Caucasian subjects and principle components for all TEDDY Subjects were calculated by projecting their data onto the estimated axes. The differences between countries (especially Finland individuals that typically have different PCs) were adjusted by the country covariate, and other population substructure (such as northern vs southern European of origin) was adjusted by PC1 and PC2. We have re-calculated the data also without correction for the first two PCs and the only marked change was that the p-value for rs4597342 in the IA analysis increased from 0.0406 to 0.0519. Therefore, we present the data with adjustment for the first two PCs throughout this publication. A total of 4850 families participated with only one child, 297 families with 2 children, and 10 families with 3 children. A robust variance estimate was used to account for the dependence within families in the Cox proportional hazard model^40^.

The secondary analysis was to study the tentative interaction between non-HLA SNPs and HLA-genotype and also country-specific association of the SNPs with the time to development of persistent confirmed IA/type 1 diabetes.

The strength and directions of associations were denoted by hazard ratios (HR) with 95% confidence intervals (95% CI). P-values less than 0.05 were considered to indicate nominal statistical significance. Significance after Bonferroni correction was achieved when p-values were less than 0.00333 (0.05/15) in the entire cohort and less than 0.000833 (0.05/60) in the HLA-specific analyses. Statistical analyses were performed using the Statistical Analytical Software (Version 9.3, SAS Institute, Cary, NC). Quality control analyses were performed using PLINK (http://pngu.mgh.harvard.edu/purcell/plink)^41^. The PCA was performed using EIGENSTRAT software^42^.

## Additional Information

**How to cite this article**: Törn, C. *et al*. Complement gene variants in relation to autoantibodies to beta cell specific antigens and type 1 diabetes in the TEDDY Study. *Sci. Rep.* **6**, 27887; doi: 10.1038/srep27887 (2016).

## Supplementary Material

Supplementary Information

## Figures and Tables

**Table 1:** Characteristics of subjects by the status of islet autoimmunity (IA) and type 1 diabetes (T1D) in The Environmental Determinants of Diabetes in the Young (TEDDY) study.

	**Subjects who developed IA**	**Subjects who did not develop IA**	**Subjects who developed T1D**	**Subjects who did not develop T1D**
*Number of subjects (n)*	413	5061	115	5359
Age at first IA, T1D or the most recent visit (months)				
Median	27.9	69.9	51.4	72.9
IQR	15.4–47.8	49.7–89.5	29.2–67.5	54.7–91.1
Country n (%)				
Finland	125 (30.3)	1327 (26.2)	41 (35.7)	1411 (26.3)
Germany	16 (3.9)	235 (4.6)	6 (5.2)	245 (4.6)
Sweden	159 (38.5)	1746 (34.5)	39 (33.9)	1866 (34.8)
US	113 (27.3)	1753 (34.7)	29 (25.2)	1837 (34.3)
High-risk HLA-DR-DQ genotype n (%)				
DR3/4	218 (52.8)	1986 (39.2)	65 (56.5)	2139 (39.9)
DR4/4	78 (18.9)	1008 (19.9)	19 (16.5)	1067 (19.9)
DR4/8	66 (16.0)	900 (17.8)	16 (13.9)	950 (17.7)
DR3/3	51 (12.3)	1167 (23.1)	15 (13.1)	1203 (22.5)
Gender n (%)				
Female	170 (41.2)	2487 (49.1)	50 (43.5)	2607 (48.7)

**Table 2:** Adjusted hazard ratios (HR) and p-values from the Cox proportional HR models in the analysis of time-to the development of islet autoimmunity (IA) and the analysis of time to the development of type 1 diabetes respectively.

	**IA**		**Type 1 diabetes**	
	**HR (95% CI)**	**p**	**HR (95% CI)**	**p**
Country (Reference = US)				
Finland	1.31 (1.01–1.69)	0.045	1.61 (0.99–2.62)	0.054
Germany	1.17 (0.70–1.98)	0.552	1.71 (0.71–4.13)	0.231
Sweden	1.23 (0.97–1.57)	0.088	1.08 (0.66–1.75)	0.765
High-risk HLA-DR-DQ genotype (Reference = DR3/3)				
DR3/4	2.37 (1.75–3.22)	<0.001	2.28 (1.30–4.00)	0.004
DR4/4	1.74 (1.22–2.47)	0.002	1.38 (0.70–2.72)	0.353
DR4/8	1.57 (1.08–2.28)	0.018	1.17 (0.57–2.40)	0.669
Gender (Reference = Male)				
Female	0.72 (0.59–0.87)	<0.001	0.80 (0.56–1.16)	0.243

Each of the two models had covariates for country of residence, HLA-DR-DQ genotype and gender.

**Table 3:** Primary statistical analysis of islet autoimmunity (IA).

**Chr**	**SNP**	Gene ofinterest	Minorallele	**MAF**	HR (95% CI) IA positivesubjects (n = 413) vs subjectswith no IA (n = 5061)	**p**
1	rs665691	*C1qC*	G	0.44829	0.98 (0.85–1.12)	0.7450
1	rs17615	*CD21*	A	0.29459	1.02 (0.88–1.19)	0.7638
1	rs1048971	*CD21*	A	0.34597	1.02 (0.88–1.18)	0.8277
1	rs4308977	*CD21*	C	0.29010	1.02 (0.88–1.19)	0.7540
1	rs4844573	*C4BPA*	C	0.37678	0.93 (0.81–1.07)	0.3332
6	rs9332739	*C2*	C	0.06065	1.04 (0.79–1.38)	0.7733
6	rs2857009	*C4*	C	0.40608	0.98 (0.82–1.18)	0.8539
9	rs10818488	*C5*	A	0.45661	1.11 (0.98–1.27)	0.1129
9	rs7029078	*C5*	G	0.37450	1.03 (0.90–1.18)	0.6711
**16**	**rs1143678**	***ITGAM***	**T**	**0.14770**	**0.80 (0.66–0.98)**	**0.0324**
**16**	**rs1143683**	***ITGAM***	**T**	**0.14773**	**0.80 (0.65–0.98)**	**0.0301**
16	rs1143679	*ITGAM*	A	0.11538	0.82 (0.66–1.01)	0.0667
**16**	**rs4597342**	***ITGAM***	**T**	**0.33638**	**1.16 (1.01–1.32)**	**0.0406**
19	rs2230199	*C3*	C	0.19865	0.94 (0.79–1.12)	0.4870
19	rs7951	*C3*	A	0.08916	0.90 (0.70–1.15)	0.3918

A total of 15 SNPs on the ImmunoChip were interrogated in The Environmental Determinants of Diabetes in the Young (TEDDY) study in 413 subjects positive for any IA versus 5061 autoantibody negative subjects. Subjects eligible for TEDDY carried any of the high risk HLA-DR-DQ genotypes. The minor allele frequency (MAF) was calculated from all subjects (n = 5474). Proportional hazard models were adjusted for HLA genotypes, sex and country of residence and population stratification. The first two principal components resulting from a principal components analysis of the US participants were included in the model to adjust for population stratification. First degree relatives were excluded from the analyses. Hazard ratios (HR) and 95% confidence intervals (95% CI) were estimated in this time-to-event analysis. A robust variance estimate was used to account for the dependence within families in the models. The factors indicating nominal significant risk or protection are indicated in bold. For a Chi-square value to remain significant after Bonferroni correction for multiple comparisons of 15 SNPs it must be less than 0.00333.

Abbreviations: CD21: C3D-receptor, complement receptor type 2, EBV-receptor ITGAM: Integrin alpha M = MAC1 or complement receptor 3, C4BPA: C4 binding protein alpha-chain.

**Table 4:** Primary statistical analysis of type 1 diabetes.

**Chr**	**SNP**	Gene ofinterest	Minorallele	**MAF**	HR (95% CI) Subjects withT1D (n = 115) vs subjectswithout T1D (n = 5359)	**p**
1	rs665691	*C1qC*	G	0.44829	1.10 (0.86–1.41)	0.4540
1	rs17615	*CD21*	A	0.29459	1.24 (0.94–1.65)	0.1296
1	rs1048971	*CD21*	A	0.34597	1.14 (0.87–1.50)	0.3333
1	rs4308977	*CD21*	C	0.29010	1.23 (0.93–1.62)	0.1499
1	rs4844573	*C4BPA*	C	0.37678	0.82 (0.62–1.08)	0.1635
6	rs9332739	*C2*	C	0.06065	1.08 (0.64–1.80)	0.7848
6	rs2857009	*C4*	C	0.40608	1.02 (0.73–1.44)	0.8984
9	rs10818488	*C5*	A	0.45661	1.15 (0.90–1.46)	0.2693
9	rs7029078	*C5*	G	0.37450	1.12 (0.86–1.47)	0.3954
16	rs1143678	*ITGAM*	T	0.14770	0.84 (0.59–1.20)	0.3447
16	rs1143683	*ITGAM*	T	0.14773	0.84 (0.58–1.20)	0.3363
16	rs1143679	*ITGAM*	A	0.11538	0.66 (0.43–1.04)	0.0709
16	rs4597342	*ITGAM*	T	0.33638	1.05 (0.81–1.37)	0.6935
19	rs2230199	*C3*	C	0.19865	1.30 (0.95–1.79)	0.1019
19	rs7951	*C3*	A	0.08916	0.90 (0.56–1.44)	0.6591

A total of 15 SNPs on the ImmunoChip were interrogated in The Environmental Determinants of Diabetes in the Young (TEDDY) study in 115 subjects that had developed type 1 diabetes versus all other subjects (n = 5359). Subjects eligible for TEDDY carried any of the high risk HLA-DR-DQ genotypes. The minor allele frequency (MAF) was calculated from all subjects (n = 5474). Proportional hazard models were adjusted for HLA genotypes, sex and country of residence and population stratification. The first two principal components resulting from a principal components analysis of the US participants were included in the model to adjust for population stratification. First degree relatives were excluded from the analyses. Hazard ratios (HR) and 95% confidence intervals (95% CI) were estimated in this time-to-event analysis. A robust variance estimate was used to account for the dependence within families in the models. For a Chi-square value to remain significant after Bonferroni correction for multiple comparisons of 15 SNPs it must be less than 0.00333.

Abbreviations:

CD21: C3D-receptor, complement receptor type 2, EBV-receptor, ITGAM: Integrin alpha M = MAC1 or complement receptor 3, C4BPA: C4 binding protein alpha chain.

**Table 5:** Display of high risk HLA-genotypes constituting the criteria for eligibility for first degree relatives and children from the general population into The Environmental Determinants of Diabetes in the Young (TEDDY).

Code inTEDDY	**Genotypes**	**Abbreviation**	Generalpopulation
A	DR3-DQA1*05:01-DQB1*02:01/DR4-DQA1*03:0X-DQB1*03:02^§^	DR3/4	Yes
B	DR4-DQA1*03:0X-DQB1*03:02^§^/DR4-DQA1*03:0X-DQB1*03:02^§^	DR4/4	Yes
C	DR4-DQA1*03:0X-DQB1*03:02^§^/DR8-DQA1*04:01-DQB1*04:02	DR4/8	Yes
D	DR3-DQA1*05:01-DQB1*02:01/DR3-DQA1*05:01-DQB1*02:01	DR3/3	Yes
E	DR4-DQA1*03:0X-DQB1*03:02^§^/DR4-DQA1*03:0X-DQB1*02:01	DR4/4	No
F	DR4-DQA1*03:0X-DQB1*03:02^§^/DR1^#^-DQA1*01:01-DQB1*05:01	DR4/1	No
G	DR4-DQA1*03:0X-DQB1*03:02^§^/DR13-DQA1*01:02-DQB1*06:04	DR4/13	No
H	DR4-DQA1*03:0X-DQB1*03:02/DR4-DQA1*03:01-DQB1*03:04	DR4/4	No
I	DR4-DQA1*03:0X-DQB1*03:02^§^/DR9-DQA1*03:01-DQB1*03:03	DR4/9	No
J	DR3-DQA1*05:01-DQB1*02:01/DR9-DQA1*03:01-DQB1*03:03	DR3/9	No

^§^DR4 subtyping was performed to exclude children from the general population with DRB1*04:03. DQB1*03:04 also qualified for inclusion into the Environmental Determinants in the Young. Subtyping was not done to distinguish DQB1*02:0X and DQA1*03:0X subtypes. ^#^In this DQB1*05:01 haplotype, DR10 had to be excluded, only DR1 was eligible. The asterisk (*) indicate that the alignment is unknown at any point.
