# Supplementary material for: Complement gene variants in relation to autoantibodies to beta cell specific antigens and type 1 diabetes in the TEDDY Study
Source: Sci Rep. 2016 Jun 16;6:27887. doi: 10.1038/srep27887 (PMC4910045; doi:10.1038/srep27887)
Supplement: Supplementary Information [file srep27887-s1.pdf]

Online Appendix. The explicit values of the HR (95%CI) for type 1 diabetes in the HLA stratified analyses presented in Figure 1 in:

Complement gene variants in relation to autoantibodies to beta cell specific antigens and type 1 diabetes in the TEDDY Study

Carina Törn[1], Xiang Liu[2], William Hagopian[3], Åke Lernmark[1], Olli Simell[4], Marian Rewers[5], Anette-G Ziegler[6], Desmond Schatz[7], Beena Akolkar[8], Suna Onengut-Gumuscu[9], Wei-Min Chen[9], Jorma Toppari[4,10], Juha Mykkänen[4, 10], Jorma Ilonen[10], Stephen S Rich[9], Jin-Xiong She[11], Ashok Sharma[11], Andrea Steck[5], Jeffrey Krischer[2] and the TEDDY Study Group*.

1 Department of Clinical Sciences, Lund University/CRC, Malmö, Sweden
2 Health Informatics Institute, Morsani College of Medicine, University of South Florida, Tampa, FL, USA
3 Pacific Northwest Diabetes Research Institute, Seattle, WA, USA
4 Department of Pediatrics, Turku University Hospital, Turku, Finland
5 Barbara Davis Center for Childhood Diabetes, University of Colorado, Aurora, CO, USA
6 Institute of Diabetes Research, Helmholtz Zentrum, München, and Klinikum rechts der Isar, Technische Universität München, and Forschergruppe Diabetes e. V., Neuherberg, Germany
7 Department of Pediatrics, University of Florida, Gainesville, FL, USA
8 National Institutes of Diabetes & Digestive & Kidney Disorders, Bethesda, MD, USA
9 Center for Public Health Genomic, University of Virginia, Charlottesville, VA, USA
10 Departments of Physiology and Pediatrics, University of Turku, Turku, Finland
11 Center for Biotechnology and Genomic Medicine, Medical College of Georgia, Augusta University, Augusta, GA, USA

HLA	SNP	ProbChisq	HazardRatio	HRLowerCL	HRUpperCL
DR3/3	rs1048971_A	0.7345	0.856	0.348	2.106
DR3/3	rs10818488_A	0.9489	1.023	0.516	2.025
DR3/3	rs1143678_A	0.9724	0.983	0.370	2.611
DR3/3	rs1143679_A	0.9110	0.936	0.292	2.999
DR3/3	rs1143683_A	0.9525	0.971	0.364	2.587
DR3/3	rs17615_A	0.8670	0.930	0.400	2.166
DR3/3	rs2230199_C	0.1924	0.477	0.156	1.452
DR3/3	rs2857009_C	0.7172	0.679	0.083	5.521
DR3/3	rs4308977_G	0.8792	0.937	0.403	2.176
DR3/3	rs4597342_A	0.6244	1.170	0.625	2.190
DR3/3	rs4844573_G	0.1146	1.866	0.860	4.050
DR3/3	rs665691_C	0.8336	0.918	0.413	2.040
DR3/3	rs7029078_G	0.6136	1.199	0.593	2.427
DR3/3	rs7951_A	0.4930	0.620	0.158	2.435
DR3/3	rs9332739_C	<.0001	0.000	0.000	0.000
DR3/4	rs1048971_A	0.1684	1.283	0.900	1.828
DR3/4	rs10818488_A	0.5967	1.089	0.794	1.494
DR3/4	rs1143678_A	0.3863	0.796	0.474	1.335
DR3/4	rs1143679_A	0.1495	0.640	0.349	1.174
DR3/4	rs1143683_A	0.3837	0.795	0.474	1.333
DR3/4	rs17615_A	0.0252	1.524	1.054	2.204
DR3/4	rs2230199_C	0.3396	1.228	0.806	1.871
DR3/4	rs2857009_C	0.5509	1.150	0.726	1.820
DR3/4	rs4308977_G	0.0588	1.425	0.987	2.058
DR3/4	rs4597342_A	0.7997	1.047	0.735	1.491
DR3/4	rs4844573_G	0.0169	0.628	0.429	0.920
DR3/4	rs665691_C	0.3754	1.173	0.824	1.668
DR3/4	rs7029078_G	0.6646	1.082	0.757	1.548
DR3/4	rs7951_A	0.9486	0.979	0.518	1.851
DR3/4	rs9332739_C	0.6070	1.203	0.595	2.433
DR4/4	rs1048971_A	0.9738	1.011	0.541	1.886
DR4/4	rs10818488_A	0.0733	1.675	0.952	2.945
DR4/4	rs1143678_A	0.8365	0.925	0.442	1.935
DR4/4	rs1143679_A	0.3145	0.571	0.192	1.702
DR4/4	rs1143683_A	0.8341	0.924	0.442	1.933
DR4/4	rs17615_A	0.5713	0.812	0.394	1.671
DR4/4	rs2230199_C	0.0002	3.200	1.750	5.851
DR4/4	rs2857009_C	0.9028	0.957	0.472	1.942
DR4/4	rs4308977_G	0.8859	0.952	0.489	1.856
DR4/4	rs4597342_A	0.9653	0.986	0.512	1.898
DR4/4	rs4844573_G	0.5362	1.216	0.654	2.262
DR4/4	rs665691_C	0.8878	1.041	0.596	1.820
DR4/4	rs7029078_G	0.3024	1.389	0.744	2.593
DR4/4	rs7951_A	0.8911	0.922	0.286	2.966
DR4/4	rs9332739_C	0.6760	1.190	0.526	2.691
DR4/8	rs1048971_A	0.9820	1.009	0.455	2.236
DR4/8	rs10818488_A	0.8802	0.939	0.417	2.118
DR4/8	rs1143678_A	0.7783	0.871	0.334	2.273
DR4/8	rs1143679_A	0.6221	0.756	0.249	2.297

DR4/8	rs1143683_A	0.7716	0.867	0.332	2.266
DR4/8	rs17615_A	0.9519	0.975	0.428	2.219
DR4/8	rs2230199_C	0.9470	0.972	0.419	2.256
DR4/8	rs2857009_C	0.7339	0.896	0.477	1.684
DR4/8	rs4308977_G	0.9823	0.991	0.435	2.255
DR4/8	rs4597342_A	0.9602	0.982	0.484	1.995
DR4/8	rs4844573_G	0.1782	0.670	0.373	1.201
DR4/8	rs665691_C	0.4927	1.151	0.770	1.721
DR4/8	rs7029078_G	0.4821	0.762	0.356	1.628
DR4/8	rs7951_A	0.8030	0.859	0.259	2.845
DR4/8	rs9332739_C	0.5119	0.519	0.073	3.680
